# Predicting Treatment Responses in Patients With Osteoarthritis: Results From Two Phase Ill Tanezumab Randomized Clinical Trials

**DOI:** 10.1002/cpt.2842

**Published:** 2023-01-31

**Authors:** Luana Colloca, Robert H. Dworkin, John T. Farrar, Leslie Tive, Jiyue Yang, Lars Viktrup, Gorana Dasic, Christine R. West, Ed Whalen, Mark T. Brown, Steven A. Gilbert, Kenneth M. Verburg

**Affiliations:** 1 Department of Pain and Translational Symptom Science, Placebo Beyond Opinions Center, School of Nursing, University of Maryland, Baltimore, Maryland, USA; 2 University of Rochester, Rochester, New York, USA; 3 University of Pennsylvania, Philadelphia, Pennsylvania, USA; 4 Pfizer Inc, New York, New York, USA; 5 Pfizer Inc, Groton, Connecticut, USA; 6 Eli Lilly and Co, Indianapolis, Indiana, USA.

## Abstract

Prediction of treatment responses is essential to move forward translational science. Our question was to identify patient-based variables that predicted responses to treatments. We conducted secondary analyses on pooled data from two randomized phase Ill clinical trials (NCT02697773 and NCT02709486) conducted in participants with moderate to severe osteoarthritis randomized to subcutaneous placebo (*n* = 514) or tanezumab 2.5 mg (*n* = 514). We used gradient boosted regression trees to identify variables that predicted Western Ontario and McMaster Universities Osteoarthritis Index (WOMAC) Pain subscale scores at Week 16 and marginal plots to determine the directional relationship between each variable category and responses to placebo or tanezumab within the models. We also used Virtual Twins models to identify potential subgroups of response to the active treatment vs. placebo. We found that responses to placebo were predicted by baseline WOMAC Physical Function, baseline WOMAC Pain, the radiographic classification of the index joint, and the standard deviation of diary pain scores at baseline. In contrast, baseline WOMAC Pain along with failure of prior medications, duration of disease, and standard deviation of diary pain scores at baseline were predictive of tanezumab responses as expressed by the WOMAC Pain scores at Week 16. Those who responded to tanezumab vs. placebo were identified based on the radiographic classification of the index joint and either age or smoking status. These secondary-data analyses identified distinct and common patient-based variables to predict response to placebo or tanezumab. These findings will inform the design of future clinical trials, helping to move forward clinical pharmacology and translational science.

The development of novel therapeutics in clinical pharmacology and medicine requires study designs that have sufficient assay sensitivity and reproducibility to detect genuine differences between the study treatments and placebo across multiple studies. Well-conducted studies of first-line treatments with proven efficacy for chronic pain conditions have failed to demonstrate statistically significant efficacy vs. placebo.^1–3^ One of the key factors that is thought to contribute to the lack of statistically significant efficacy outcomes in these studies is the improvement observed in patients who received placebo. A recent meta-analysis of chronic pain studies conducted in the United States reported an increase in placebo responses relative to responses to active treatment over time.^4^

Several studies have examined patient characteristics and aspects of trial design or implementation that might be important factors in determining patients’ response to placebo or potential or established treatments. Patient-based variables including sex, race, concomitant conditions, and medications, as well as individual patient expectations and prior therapeutic experience have all been shown to influence both treatment and placebo responses in chronic pain studies. ^5–8^

Another factor that might contribute to differences in response is the variability in patients’ response to treatment, also known as treatment effect heterogeneity.^9^ The ability to identify patients based on differential response to treatment vs. placebo would greatly improve clinical trial design, assay sensitivity, and outcomes and has the potential to contribute to the development of precision pain medicine. ^10^ Factors associated with treatment effect heterogeneity include baseline pain variability, use of rescue medication, and excessive variability of pain ratings.^11–14^

In this retrospective analysis, we used results from two recent randomized placebo-controlled phase III studies of subcutaneous (SC) tanezumab, an antibody against nerve growth factor that has been in the pipeline as a potential pain treatment for patients with osteoarthritis (OA). ^15,16^ On October 26, 2021, Pfizer Inc. and Eli Lilly and Company announced discontinuation of the global clinical development program as a result of the outcomes of regulatory reviews of tanezumab for the treatment of OA pain by the US Food and Drug Administration and European Medicines Agency. ^17,18^ Yet, we conducted retrospective analyses of these two tanezumab studies to create predictive models for patient-level variables that contributed most to postbaseline scores for the Western Ontario and McMaster Universities Osteoarthritis Index (WOMAC^a^) Pain efficacy subscale.

## MATERIALS AND METHODS

### Study design of pooled trials

**Figure 1:**
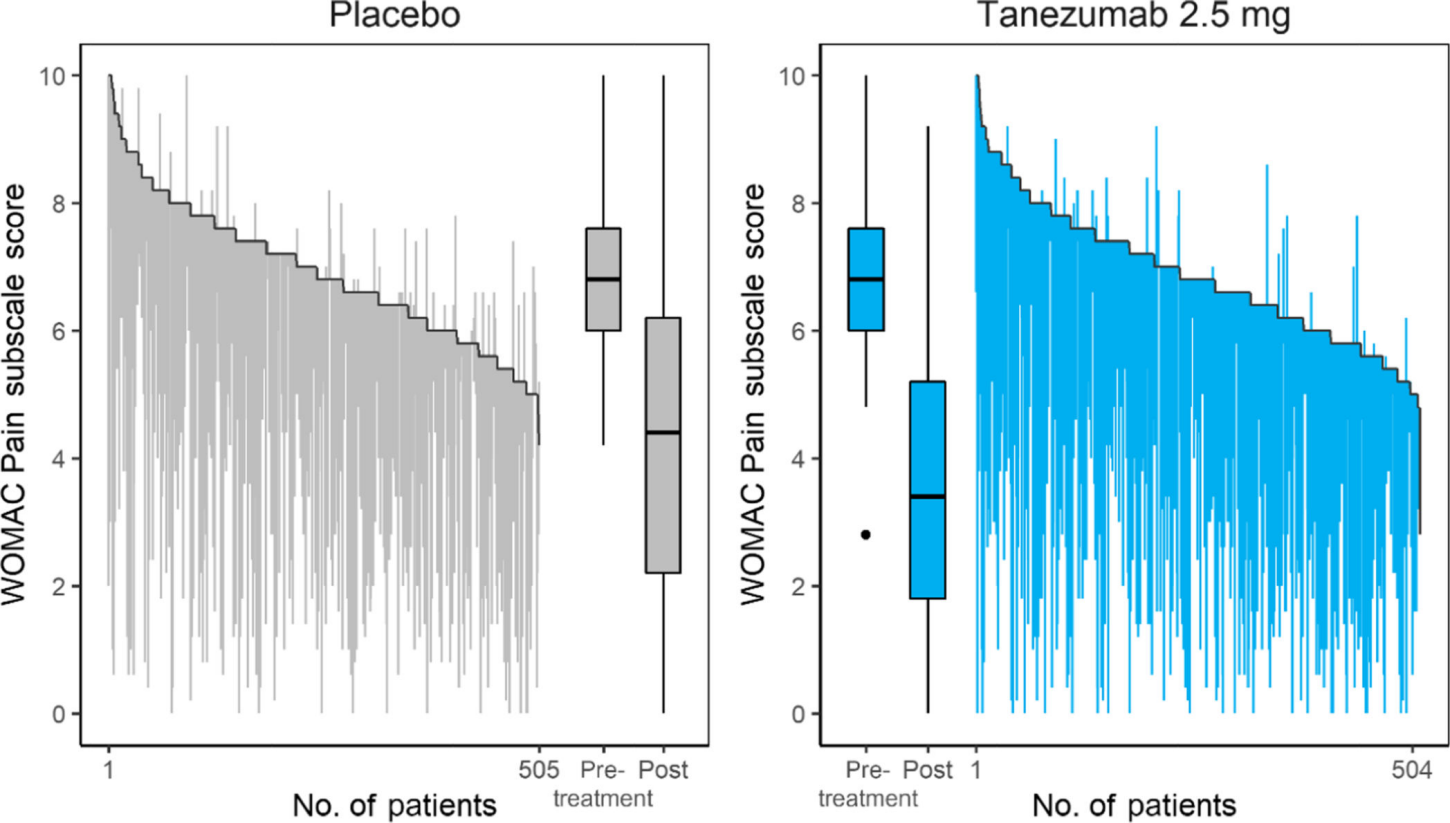
The black lines represent baseline values for each individual patient, sorted by severity. Individual colored lines represent the change from baseline at Week 16 for each individual patient (colored lines extending below the black line indicate lower score and, thus, improvement at Week 16). The pretreatment (baseline) and posttreatment (Week 16) boxplots show median (middle horizontal line in the box), and quartiles 1 and 3 (bottom and top lines of the box). Lines extend from the box to the smallest and largest observations no further than 1.5 times the interquartile range from quartile 1 and quartile 3, respectively, and any data beyond this range are plotted individually. WOMAC Pain subscale ranges from 0 = no pain to 10 = extreme pain. WOMAC, Western Ontario and McMaster Universities Osteoarthritis Index.

Data were pooled from two phase III double-blind, placebo-controlled clinical trials of SC tanezumab in patients with OA of the hip or knee conducted between 2016 and 2018. ^15,16,19^ Findings from the original trials are depicted in Figure 1. Study A4091056 (NCT02697773), hereafter referred to as study 1,056, had a ≤37-day screening period, a 16-week double-blind treatment period, and a 24-week safety follow-up period, and was conducted in North America. ^15^ During the treatment period, patients were randomized to receive 1 of 3 treatment regimens: SC placebo or tanezumab 2.5 mg at baseline and Week 8, or tanezumab 2.5 mg at baseline and 5 mg at Week 8. Study A4091057 (NCT02709486), hereafter referred to as study 1,057, had a ≤37-day screening period, a 24-week double-blind treatment period, and a 24-week safety follow-up period, and was conducted in Europe and Japan. ^16^ During the treatment period, patients were randomized to receive either SC placebo or tanezumab 2.5 mg or 5 mg at baseline and at Weeks 8 and 16.

### Study population

Full inclusion and exclusion criteria for trials 1,056 (ref. 15) and 1,057 (ref. 16) have been previously described and are included in Supplementary Materials. Briefly, key inclusion criteria in both trials were patients aged ≥18 years with a diagnosis of OA with a Kellgren - Lawrence (KL) grade ≥2 in the index hip or knee with radiographic confirmation by a central reader at screening; a WOMAC Pain subscale score of ≥5 in the index joint at screening and baseline, a WOMAC Physical Function subscale score of ≥5 at baseline, and a Patient’s Global Assessment of Osteoarthritis of “fair,” “poor,” or “very poor” at baseline, and the index joint as the most painful joint at screening with a qualifying WOMAC Pain score and KL grade.

Key exclusion criteria for both studies included a history of non-OA joint disease in the index joint, radiographic evidence of rapidly progressive OA, atrophic OA, subchondral insufficiency fractures, osteonecrosis or pathological fracture at screening, or a history of significant trauma or surgery to a knee, hip, or shoulder in the previous year.

### Key efficacy end points

WOMAC Index Version 3.1 numeric rating scale (NRS) was utilized. ^20^ WOMAC Pain and WOMAC Physical Function were coprimary end points of both studies. ^15,16^ Each subscale was assessed using an 11-point NRS (0 = no pain/difficulty to 10 = extreme pain/difficulty). The recall period for all WOMAC subscales was the prior 48 hours. The WOMAC was completed during study visits at baseline and at Weeks 2, 4, 8, 12, and 16 in both studies. In addition to the WOMAC, patients completed electronic diary assessments of pain in the index joint using an 11-point NRS (0 =no pain to 10 =worst possible pain) at approximately the same time each day.

### Statistical analyses

The analyses pooled the placebo arms from both trials (*n* = 232 in study 1,056 and *n* = 282 in study 1,057); likewise, the patients on tanezumab 2.5 mg were pooled (*n* = 231 in study 1,056 and *n* = 283 in study 1,057). WOMAC Pain scores at Week 16 were selected as the end point for these analyses, as data from this timepoint were collected in both studies. The response to study treatment was investigated in the tanezumab 2.5-mg arm, as this dose was used for the entire double-blind treatment period of both trials. ^15,16^

Patient characteristics were included as variables of interest in these analyses if there was a rationale that they might influence placebo or treatment response and there was a sufficient number of patients with that characteristic to allow the variable to be tested (Figure S1). After selection, the variables were assessed for redundancy using Spearman rank correlation matrices. The variables included in these analyses and their abbreviations are listed in Table 1.

All analyses were performed in R (https://www.r-project.org/).^21^ *P* values were reported only as descriptive statistics and were not intended to be interpreted as formal hypothesis tests. The primary predictive modeling method, which was run separately for placebo and tanezumab 2.5-mg responses, used gradient boosted trees fitted with generalized boosted models (GBMs).^22^ GBMs are a popular machine learning algorithm that have proven successful across many domains. GBMs work by creating an ensemble of small decision trees by allocating variables to nonoverlapping groups based on similar responses. They do this by using designated variable “splitting rules” (e.g., split on high vs. low baseline pain). This allows the trees to simultaneously select variables, model nonlinear relationships, and model interactions between variables by splitting on one variable and then another. Each additional tree in the sequence is designed to improve the fit of the current model using all previous trees. A cross-validation methodology is used to decide how many trees should be included in the ensemble to best predict responses on future independent data and avoid overfitting. Models were fitted assuming the data had a Gaussian distribution and the individual decision trees were allowed to include up to three-way interactions.

The predictive ability of the model for the patients in the provided data set was calculated as a percentage of variability explained by the model, termed *R*^2^. Given that this study did not have a large enough sample size to do a normal split of the data, we opted for the next best procedure which is a 10-fold cross-validation which in part does the splitting 10 times and averages across the corresponding 10 predictive models.^23^ Therefore, the estimated ability of the model to predict values for patients not included in the model was calculated as a percentage using a separate 10-fold cross-validation procedure.

Sensitivity analyses were performed by repeating the above analyses using the R package random Forest.^24^ Random forests are another ensemble method for combining multiple decision trees that fit many large but uncorrelated trees. Each tree was fitted to a bootstrap sample of the data, and a random sample of the variables was selected to create a single tree with the given bootstrap sample. Finding similar results from this methodology increased the likelihood that the primary analyses h ad not just modeled idiosyncrasies of the data that were unlikely to be replicated in future studies. A main effects linear model was also used to investigate patient-based variables that predicted postbaseline WOMAC Pain scores to compare and confirm the results of the primary analyses.

### Subgroup Identification

The primary analysis for identification of potential subgroups with enhanced response to tanezumab 2.5 mg vs. placebo was performed using a Virtual Twins strategy.^25^ This approach built upon the random forest models constructed previously to model the response of every patient to placebo and tanezumab 2.5 mg. Although the random forest methods originally were planned as a second look at the modeling of individual treatment groups, these were chosen for this combined step because of their better performance.

In the first step of the Virtual Twins analysis, predictive models for placebo and tanezumab 2.5-mg response were fitted separately with the GBM methods previously described. In the second step, the estimated individual treatment effects were calculated as the difference between a subject’s predicted response under treatment and their predicted response under control. The individual treatment effects were analyzed using model-based recursive partitioning^26^ with the R package partykit.^27^ This method partitions subjects based on their individual treatment effects using the same variables as the previously described predictive models for placebo and tanezumab. The method was used to identify subgroups with a treatment difference greater than the effect found using the linear model analyses that were initially performed. This enhancement in treatment effect was bias-corrected using a boot-strap methodology.^28^ Sensitivity analyses were performed using the random Forest package.^24^

## RESULTS

Demographics, clinical characteristics, and indicators of disease status were similar between treatment arms (Table 2).

### Predictive modeling of postbaseline WOMAC pain scores in patients who received placebo

**Figure 2:**
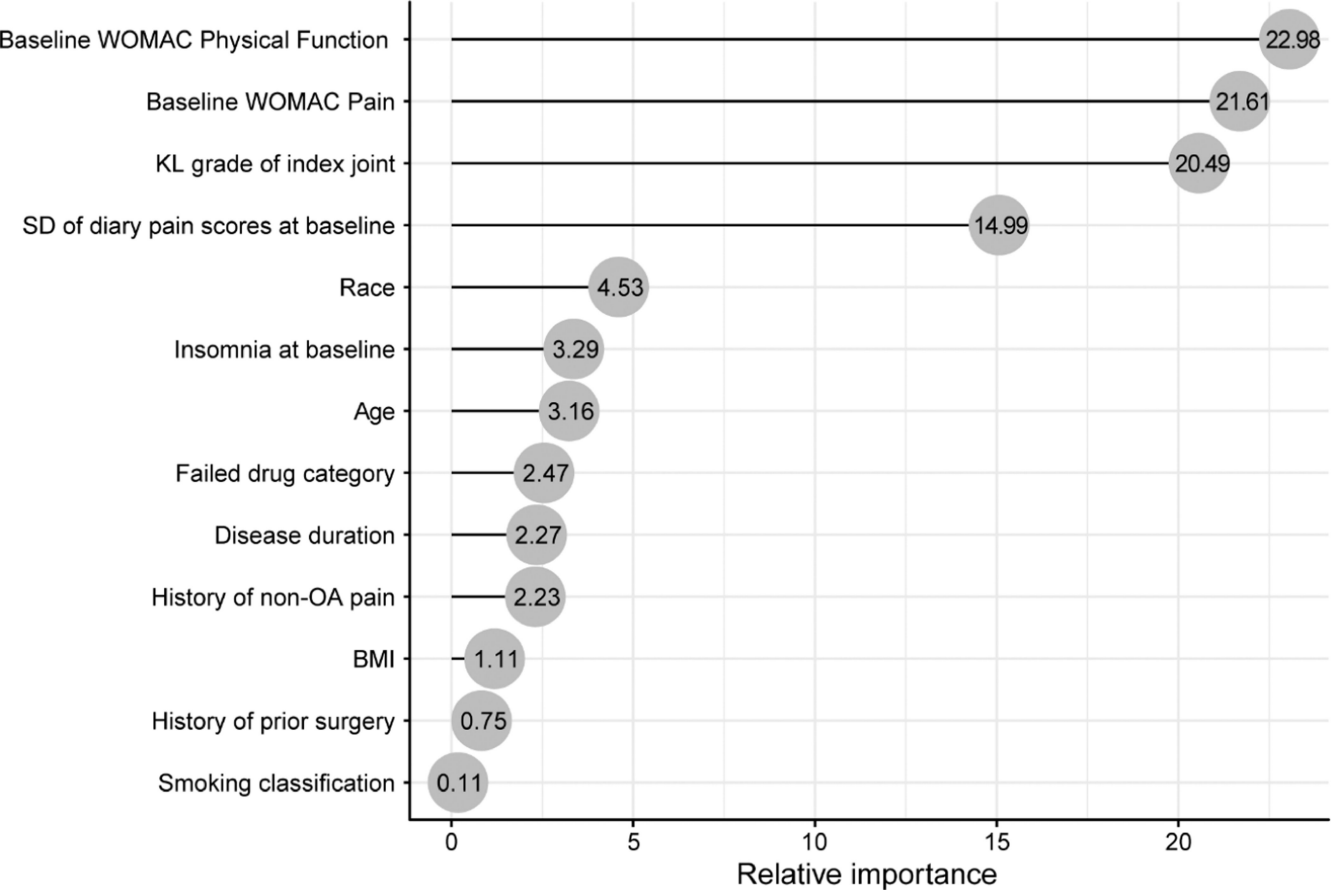
Gradient boosted regression trees for variables predictive of WOMAC Pain scores at Week 16 in the placebo arm. We used gradient boosted regression trees to identify the variables that contributed most to WOMAC Pain scores at Week 16 in patients who received placebo. These analyses identified baseline WOMAC Physical Function, baseline WOMAC Pain, the KL grade of the index joint, and the SD of diary pain scores at baseline as the most important predictors of WOMAC Pain scores at Week 16. The overall predictive ability of the model for the data provided was 22%, and the estimated predictive ability for new data was 9.8%. BMI, body mass index; KL, Kellgren-Lawrence; OA, osteoarthritis; SD, standard deviation; WOMAC, Western Ontario and McMaster Universities Osteoarthritis Index.

Analysis of the placebo data set using gradient boosted tree modeling identified baseline WOMAC Physical Function, baseline WOMAC Pain, the KL grade of the index joint, and the standard deviation of baseline diary pain scores as the most important predictors of WOMAC Pain scores at Week 16 (Figure 2). The model explained 22% of the variability in values for the patients included in the data set used to build the model. The estimated ability of model to predict values for patients not included in the model was 9.8%. The variables identified by gradient boosted tree modeling were also identified as important predictors of WOMAC Pain scores at Week 16 in a main effects linear regression model (Figure S2). In particular, patients with higher baseline WOMAC Physical Function scores had higher WOMAC Pain scores at Week 16, compared with those who had lower baseline WOMAC Physical Function scores (Figure S3A). Patients with higher baseline WOMAC Pain scores had higher WOMAC Pain scores at Week 16, compared with those who had lower baseline WOMAC Pain scores (Figure S3B). Patients with a KL grade of 4 in the index joint had higher WOMAC Pain scores at Week 16, compared with those with KL grades 2–3 (Figure S3C). Patients with more variable diary pain scores at baseline indicated by higher standard deviations had lower WOMAC Pain scores at Week 16, compared with those with lower standard deviations of baseline diary scores (Figure S3D).

### Predictive modeling of postbaseline WOMAC pain scores in patients who received tanezumab 2.5 mg

**Figure 3:**
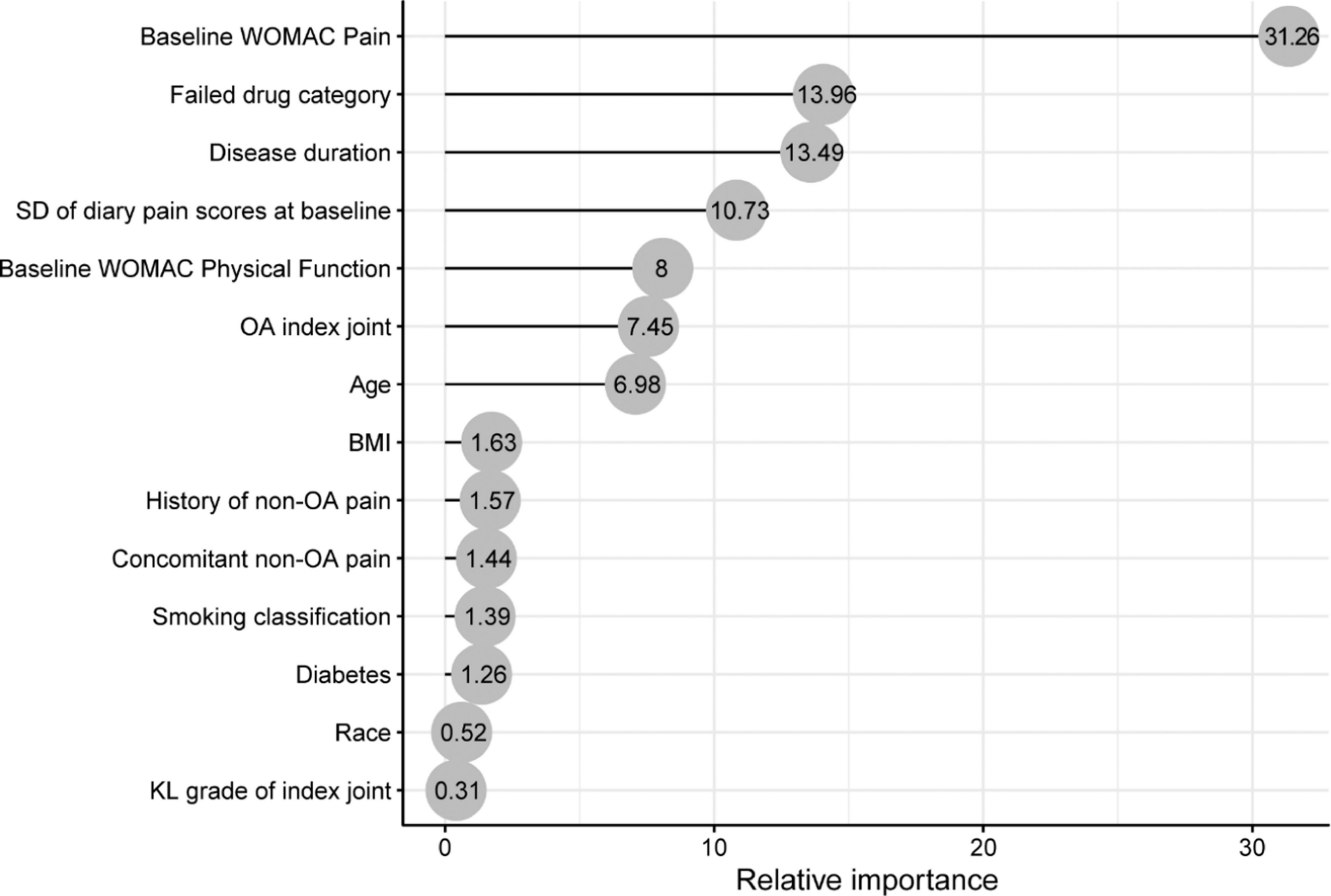
Gradient boosted regression trees for variables predictive of WOMAC Pain scores at Week 16 in the tanezumab 2.5-mg arm. We used gradient boosted regression trees to identify the variables that contributed most to WOMAC Pain scores at Week 16 in patients who received tanezumab 2.5 mg. These analyses identified baseline WOMAC Pain, failure of prior medications, duration of disease, and the SD of diary pain scores at baseline as the most important predictors of WOMAC Pain scores at Week 16. The overall predictive ability of the model for the data provided was 22.9%, and the estimated predictive ability for new data was 6.4%. BMI, body mass index; KL, Kellgren-Lawrence; OA, osteoarthritis; SD, standard deviation; WOMAC, Western Ontario and McMaster Universities Osteoarthritis Index.

Analysis of the tanezumab data set using gradient boosted tree modeling identified baseline WOMAC Pain scores, failed drug category, duration of disease, and the standard deviation of diary pain scores at baseline as the most important predictors of WOMAC Pain scores at Week 16 in this treatment arm (Figure 3). The overall predictive ability of the model for the provided data set was 22.9%. The estimated predictive ability of the model for new data was 6.4%. The variables identified by gradient boosted tree modeling were also identified among the most important predictors of WOMAC Pain scores at Week 16 by a main effects linear regression model (Figure S4). Specifically, patients with higher baseline WOMAC Pain scores had higher WOMAC Pain scores at Week 16, compared with those who had lower baseline WOMAC Pain scores (Figure S5A). Patients who had contraindication to NSAID, opioids, and/or tramadol had lower WOMAC Pain scores at Week 16 compared with those who had all other categories of prior drug failures (Figure S5B). With the exception of newly diagnosed patients, those with durations of disease longer than ~ 10 years had higher WOMAC Pain scores at Week 16 than those with comparatively shorter durations of disease (Figure S5C). Patients with higher standard deviations of diary pain scores at baseline had lower WOMAC Pain scores at Week 16, compared with those with lower standard deviations of diary pain scores at baseline (Figure S5D).

### Identification of patients subgroups for enhanced treatment effects

Based on the results of gradient boosted tree models for placebo and ranezumab treatment arms, identification of subgroups with enhanced treatment effects was performed using Virtual Twins. These analyses aimed to identify subgroups of patients in which the enhanced treatment effect of tanezumab 2.5 mg vs. placebo was greater than that produced from a linear model using the same data set (−0.6872).

**Figure 4:**
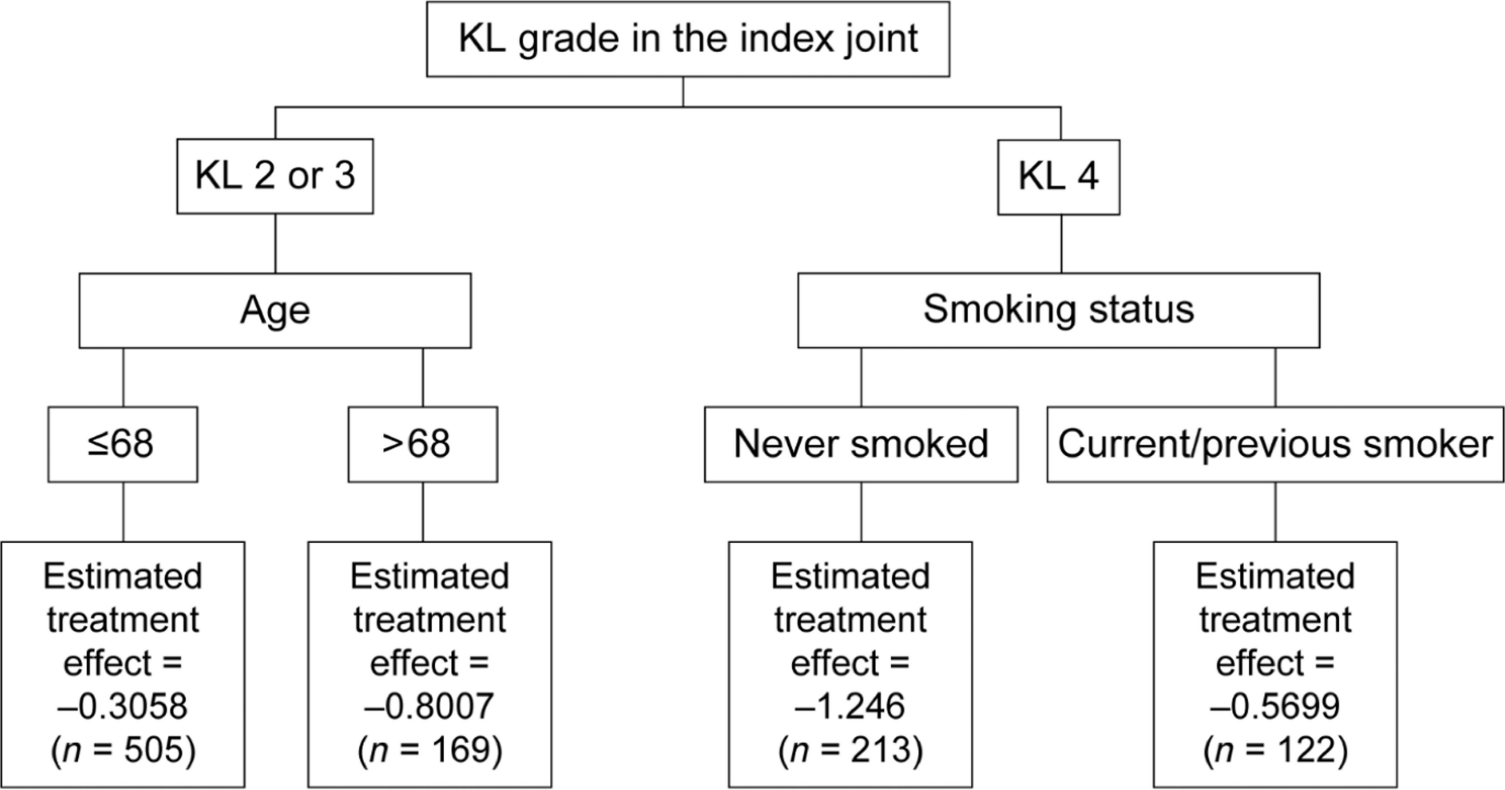
Subgroup identification with enhanced response to tanezumab 2.5 mg vs. placebo. Virtual Twins analyses using a random forest model split the data by KL grade in the index joint and then age or tobacco class. Two subgroups of patients were predicted to have an increased response to tanezumab vs. placebo that was greater than that estimated by the linear model. The first subgroup contained patients aged >68 years with a KL grade of 2 or 3 in the index joint. The second subgroup contained patients who had never smoked with a KL grade of 4 in the index joint. Correction of these analyses indicated that optimism bias of the method was smaller than the enhanced treatment effect, suggesting that these findings may generalize to new data. For failed drug categories in Panel (a), Cat A contained patients with contraindication to NSAID, opioid, and/or tramadol, inadequate pain relief for all three classes, inadequate pain relief for only NSAID and/or tramadol, no contraindication but intolerability to NSAID, opioid, and/or tramadol. Cat B contained patients with inadequate pain relief for only NSAID and opioid, no contraindication, intolerability to NSAID and/or tramadol/opioid and unwilling to take tramadol/opioid. KL, Kellgren-Lawrence; Cat, Category; NSAID, nonsteroidal anti-inflammatory drug; WOMAC, Western Ontario and McMaster Universities Osteoarthritis Index.

Applying the randomForest package to the Virtual Twins analysis split the data first by KL grade of the index joint (2 or 3 vs. 4; Figure 4). Dara for patients with a KL grade of 2 or 3 were further split by age (≤68 vs. >68 years). Data for patients with a KL grade of 4 in the index joint were further split by smoking classification (never smoked vs. current or previous smoker). In this model, two subgroups of patients were predicted to have an increased response to tanezumab vs. placebo that was greater than that estimated by the linear model. The first subgroup contained patients aged >68 years with a KL grade of 2 or 3 in the index joint (Figure 4). The second subgroup contained patients with a KL grade of 4 in the index joint who had never smoked (Figure 4). Bootstrap correction of these analyses indicated that the bias estimate of the model (−0.1759) was smaller than the average enhanced treatment effect of the two subgroups (−0.4299), indicating that these findings may generalize to new data with an effect of approximately −0.25. Additional negative results for the identification of subgroups are presented in Figure S6.

## DISCUSSION

This study identified patient-based variables that contributed to postbaseline WOMAC Pain scores in patients with OA who received placebo or ranezumab 2.5 mg and identified subgroups of patients with enhanced tanezumab and placebo treatment responses. Predictive models created separately for placebo and tanezumab 2.5 mg arms both identified baseline WOMAC Pain score and the standard deviation of diary pain scores at baseline as important variables associated with WOMAC Pain scores at Week 16. In both the placebo and ranezumab 2.5-mg arms, patients with higher baseline WOMAC Pain scores had higher WOMAC Pain scores at Week 16, compared with those with lower baseline scores. Baseline WOMAC Physical function scores and the KL grade of the index joint were identified as factors influencing WOMAC Pain scores at Week 16 in the placebo arm but were not important in the ranezumab model, suggesting that predictors of treatment responses differ between patients who are more likely to respond to placebo than ranezumab.

Placebo responses are complex in nature and a challenge when new treatments are under invesrigarion.^6^ Larger placebo responses have been found in chronic pain related to polyneuropathies as compared with central neuropathic pain or postherpetic neuralgia, in pain-related parallel-design trials compared with crossover designs,^2,29^ longer trial duration with higher visits, ^30,31^ and in the United States as compared with other countries.^4^ In particular, severity of the disease has been associated with placebo responses in depression^32^ and neuropathic^12^ studies. We found that *higher* baseline severity was correlated with *larger* responsiveness to placebos. Placebo-treated patients with higher baseline WOMAC Physical Function scores had higher WOMAC Pain scores at Week 16, compared with those who had lower baseline WOMAC Physical Function scores. These findings might suggest that patients with greater functional disability may have had higher expectations for positive outcomes in these studies, resulting in larger placebo responses. Placebo-treated patients with a KL grade of 4 in the index joint, indicating severe OA, had larger placebo responses in those with higher WOMAC Pain scores at Week 16 than those with KL grades of 2 or 3, indicating minimal or moderate disease, respectively.

Predictors for tanezumab were different from placebos. Duration of disease and failure of prior medications were identified as variables predictive of Week 16 WOMAC Pain scores in response to tanezumab but were not important in the placebo model. With the exception of newly diagnosed patients, those with greater than ~ 10 years of disease history had higher WOMAC Pain scores at Week 16 in response to tanezumab administration than those with less than 10 years of disease. A correlation between increased disease duration and decreased response to treatment has been previously observed in a meta-analysis of 14 randomized trials in patients with rheumatoid arthritis,^33^ suggesting that disease duration could be a factor that should be considered in trial designs. Additionally, patients with contraindications to NSAID, opioids, and/or tramadol had lower WOMAC Pain scores at Week 16 in response to tanezumab administration than those with prior failures or intolerability to those drug classes. Prior failures or intolerability to NSAID, opioids, and/or tramadol drug classes may have created lower and negative expectations, therefore jeopardizing the response toward new investigational drugs.

In our analyses, higher baseline pain variability was associated with lower WOMAC Pain scores at Week 16 in both the placebo and tanezumab arms. However, unlike the results of previous studies, ^13,14,34^ there was no evidence for an association between baseline pain variability and differences between tanezumab and placebo (i.e., assay sensitivity). Our results are in line with another recent retrospective analysis to identify predictors of placebo responses for neuropathic pain whereby baseline pain variability was not associated with placebo responses in crossover design, controlled clinical trials in patients with peripheral neuropathic pain.^35^ The explanation for these contrasting results could involve study design or treatment characteristics, for example, oral medications taken on a daily basis vs. subcutaneous tanezumab administered every 8 weeks.

Identification of patient subgroups with enhanced responses to study treatment vs. placebo could help inform trial designs and contribute to the clinical application of precision pain medicine.^36^ Our analyses identified two subgroups of patients who had an enhanced response to tanezumab vs. placebo. The first subgroup contained patients with a KL grade of 2 or 3 in the index joint and age >68 years. The second subgroup with an enhanced effect of tanezumab vs. placebo contained patients with a KL grade of 4 in the index joint who had never smoked. The average enhanced treatment effect size for these subgroups was larger than the calculated optimism bias of the model, suggesting that the subgroup findings may generalize to new data. In support of these results, baseline disease severity has been associated with treatment response in several meta-analyses.^32,37^ Smokers with chronic pain have worse pain outcomes than nonsmokers, ^38^ and smokers have also been shown to have reduced response to biologics for the treatment of rheumatoid arthritis.^39,40^ Future trials should investigate the effect of smoking on citrullination.

There are some limitations to acknowledge. A possible limitation of the study is that although the predictive modeling approaches used revealed several significant predictors of patient response to placebo or tanezumab, the amount of variability they explained within the data remains low. For the data provided, the models explained 22–22.9% of variability in patient scores and the estimated predictive ability of the models for new data was 6.4–9.8%. A possible explanation for these results is that we conducted retrospective analyses of two trials designed to investigate the efficacy and safety of tanezumab vs. placebo. Future studies designed to use the methods described here may benefit from inclusion of a greater number of OA patients and related variables screening proactively measures for outcomes of interest to enrich the patient population and consequently improve the predictive ability of the models. Another limitation is that the patient population selected for the studies analyzed might not completely represent that seen in clinical practice due to the inclusion and exclusion criteria applied. Consequently, the effectiveness of the predictors identified in a clinical care setting may require additional validation in future research.

Despite these limitations, current findings hint to the relevance of determining clinical phenotypes and hopefully, biomarkers that predict responses to treatments vs. placebos. Future applications of the predictive model being developed in this study include its translation to other trials for pain disorders and other conditions. Ideally, an extension of these findings to other conditions will help leverage clinical phenotypes (and biomarkers) to improve clinical trial designs including a consideration of the appropriateness of using the identified factors to randomize patients with the target phenotypes equally across arms and/or restrict them when a new clinical trial design is developed.

## CONCLUSIONS

Baseline WOMAC Physical Function and the KL classification of OA were predictors of response to placebo, whereas duration of disease and failure of prior medications were predictive of response to tanezumab. Subgroups of patients with enhanced response to tanezumab vs. placebo were identified based on the radiographic classification of the index joint and either age or smoking status. Further testing of the patient-based variables to identify treatment and placebo outcomes and patients’ subgroups are potentially informative. Ideally, the identification of biomarkers of treatment responses that are not influenced by placebos could help advance drug development. A better understanding of patients’ responses to placebos and study treatments in clinical trials for chronic pain conditions has the potential to improve study designs, assay sensitivity, and outcomes, contributing to the development of precision pain medicine.

## Supplementary Material

Suppl Material

## Figures and Tables

**Table 1:** Patient-based variables analyzed during the study

Characteristic	Abbreviation
Study	STUDYID
Race	RACE
Age	AGE
Sex	SEX
BMI (<25, 25 to <30, 30 to <35, and ≥35)	BMI
OA index joint (hip or knee)	JINDOVER
KL grade of index joint (2, 3, or 4)^a^	KLGRDJIN
Duration of disease	ADURN
Baseline WOMAC pain	BASE
Standard deviation of diary pain scores at baseline	DPAINSD
Baseline WOMAC Physical Function scores	WMPFUNC
History of psychiatric disorders (insomnia, depression, or anxiety)	PSYCHFL
Insomnia at baseline	INSMBFL
Diabetes (yes/no)^b^	DIAFL
Alcohol consumption (yes/no)	ALCFL
Smoking classification (nonsmoker, smoker, or ex-smoker)	TBCLASS
Concomitant non-OA pain	CMHOAFL
History of non-OA pain	NONOAFL
History of prior surgery	SURG
Failed drug category^c^	FDRGCAT
Rescue medication use	RESUSEFL

BMI, body mass index; KL, Kellgren-Lawrence; NSAID, nonsteroidal anti-inflammatory drug; OA, osteoarthritis; WOMAC, Western Ontario and McMaster Universities Osteoarthritis Index.

a Patients with KL grade <2 were included in the KL grade 2 category for modeling.

b Diabetes was defined by medical history terms from a prespecified sponsor-generated custom Medical Dictionary for Regulatory Activities (MedDRA) query and/or a screening hemoglobin A1c ≥6.5%.

c Failed drug categories were inadequate pain relief for only NSAID and/or tramadol; no contraindication but intolerability to NSAID, opioid, and/or tramadol; no contraindication, intolerability to NSAID and/or tramadol/opioid and unwilling to take tramadol/opioid; contraindication to NSAID, opioid, and/or tramadol; inadequate pain relief for all three classes; inadequate pain relief for only NSAID and opioid.

**Table 2:** Baseline demographics, clinical characteristics, and disease status

Characteristic	Placebo (*n* = 514)	Tanezumab 2.5mg (*n* = 514)
Age, mean (SD), y	62.5 (9.8)	63.2 (9.4)
Female, *n* (%)	353 (68.7)	343 (66.7)
Index joint, *n* (%)		
Hip	80 (15.6)	83 (16.1)
Knee	434 (84.4)	431 (83.9)
KL grade of index joint, *n* (%)		
0	0	2 (0.4)
1	0	1 (0.2)
2	124 (24.1)	109 (21.2)
3	221 (43.0)	232 (45.1)
4	169 (32.9)	170 (33.1)
Disease duration, mean (SD), y^a^	8.7 (8.1)	7.9 (7.8)
WOMAC pain, mean (SD)^a^	6.9 (1.1)	6.9 (1.1)
WOMAC physical function,	7.0 (1.1)	7.0 (1.0)
mean (SD)^a^		
PGA-OA, mean (SD)^a^	3.5 (0.6)	3.5 (0.6)
Diary pain, mean (SD)^a^	7 (1.4)	6.9 (1.4)
BMI, *n* (%), kg/m^2^		
<25	51 (9.9)	72 (14.0)
25 to < 30	165 (32.1)	162 (31.5)
30 to <35	168 (32.7)	172 (33.5)
≥ 35	130 (25.3)	108 (21.0)
Race, *n* (%)		
White	403 (78.4)	423 (82.3)
Black or African American	60 (11.7)	43 (8.4)
Asian	47 (9.1)	43 (8.4)
Other/Unknown	4 (0.8)	5 (1.0)
Insomnia at baseline, *n* (%)	56 (10.9)	48 (9.3)
Diabetic, *n* (%)^b^	84 (16.3)	86 (16.7)
Alcohol drinker, *n* (%)	231 (44.9)	200 (38.9)
Current smoker, *n* (%)	66 (12.8)	48 (9.3)
History of psychiatric disorders, *n* (%)^c^	109 (21.2)	118 (23.0)
Concomitant non-OA pain, *n* (%)	85 (16.5)	70 (13.6)
History of non-OA pain, *n* (%)	98 (19.1)	87 (16.9)
History of prior surgery, *n* (%)	229 (44.5)	230 (44.8)

BMI, body mass index; KL, Kellgren-Lawrence; OA, osteoarthritis; PGA-OA, patient’s global assessment of osteoarthritis; SD, standard deviation; WOMAC, Western Ontario and McMaster Universities Osteoarthritis Index.

a Disease duration was reported for 514 and 512 patients in the placebo and tanezumab 2.5-mg arms, respectively. Baseline WOMAC Pain, WOMAC Physical Function, and PGA-OA were reported for 513 patients in both arms. Baseline diary pain was reported for 506 and 508 patients in the placebo and tanezumab arms, respectively.

b Diabetes was defined by medical history terms from a prespecified sponsor-generated custom MedDRA (Medical Dictionary for Regulatory Activities) query and/or a screening hemoglobin A1c ≥6.5%.

c History of psychiatric disorders comprised insomnia, depression, and anxiety.

## Data Availability

Upon request, and subject to certain criteria, conditions and exceptions (see https://www.pfizer.com/science/clinicaI-trials/triaI-data-and-results for more information), Pfizer will provide access to individual deidentified participant data from Pfizer-sponsored global interventional clinical studies conducted for medicines, vaccines and medical devices (i) for indications that have been approved in the United States and/or Europe or (ii) in programs that have been terminated (i.e., development for all indications has been discontinued). Pfizer will also consider requests for the protocol, data dictionary, and statistical analysis plan. Data may be requested from Pfizer trials 24 months after study completion. The deidentified participant data will be made available to researchers whose proposals meet the research criteria and other conditions, and for which an exception does not apply, via a secure portal. To gain access, data requestors must enter into a data access agreement with Pfizer.

## Study Highlights

### WHAT IS THE CURRENT KNOWLEDGE ON THE TOPIC?

☑ Current knowledge in predicting placebo and active treatment responses remains in its infancy.

### WHAT QUESTION DID THIS STUDY ADDRESS?

☑ Our research question was to identify patient-based predictors of treatment (placebo and active treatment) responses with the goal to advance precision medicine and translational science.

### WHAT DOES THIS STUDY ADD TO OUR KNOWLEDGE?

☑ We found that baseline Western Ontario and McMaster Universities Osteoarthritis Index Physical Function and the Kellgren-Lawrence classification of osteoarthritis are predictors of placebo responses. On the contrary, duration of disease and failure of prior medications predicted treatment responses.

### HOW MIGHT THIS CHANGE CLINICAL PHARMACOLOGY OR TRANSLATIONAL SCIENCE?

☑ Demonstrating that predictors of placebo and treatment responses do not necessarily overlap helps to advance clinical pharmacology and efforts to improve study designs for validation of new treatments.

## Footnotes

a © 1996 Nicholas Bellamy. WOMAC® is a registered trademark of Nicholas Bellamy (CDN, EU, USA).
