# Supplementary material for: Predicting Treatment Responses in Patients With Osteoarthritis: Results From Two Phase Ill Tanezumab Randomized Clinical Trials
Source: Clin Pharmacol Ther. Author manuscript; Available in PMC 2024 Apr 8. (PMC11000258; doi:10.1002/cpt.2842)
Supplement: Suppl Material [file NIHMS1961837-supplement-Suppl_Material.docx]

SUPPLEMENTARY MATERIALS

**Predicting Treatment Responses in Patients with Osteoarthritis: Results from Two Phase 3 Tanezumab Randomized Clinical Trials**

Luana Colloca^1^, Robert H. Dworkin^2^, John T. Farrar^3^, Leslie Tive^4^ Jerry Yang^5^, Lars Viktrup^6^, Gorana Dasic^4^, Christine R. West^5^, Ed Whalen^4^, Mark T. Brown^5^, Steven A. Gilbert^4^ and Kenneth M. Verburg^5^

^1^ Department of Pain and Translational Symptom Science, School of Nursing, University of Maryland, MD, USA, ^2^University of Rochester, NY, USA, ^3^University of Pennsylvania, PA, USA, ^4^Pfizer Inc, NY, USA, ^5^Pfizer Inc, CT, USA, ^6^Eli Lilly and Co., IN, USA,

**MATERIALS AND METHODS**

The protocols for each study were approved by an institutional review board or independent ethics committee at each participating investigational center. The studies were conducted in compliance with the ethical principles of the Declaration of Helsinki and Good Clinical Practice Guidelines. All patients provided written informed consent before entering the trials.

**Additional inclusion and exclusion criteria**

- Inclusion criteria: Patients also had a documented history of insufficient pain relief from acetaminophen; and insufficient pain relief or inability to tolerate or contraindication to nonsteroidal anti-inflammatory drugs (NSAIDs); and insufficient relief from, inability to tolerate or contraindication to either tramadol or other opioids, or unwillingness to take opioids.
- Exclusion criteria: Patients with fibromyalgia or other moderate-to-severe pain that may confound assessments of OA pain; a history, diagnosis, or signs and symptoms of clinically significant neurological disease or psychiatric disorder; or a known history of alcohol, analgesic, or drug abuse within 2 years of screening were also excluded.

**Missing data**

Of the 1028 patients, 4 in the placebo arm, and 3 in the tanezumab 2.5mg arm of study 1056, were omitted from analyses due to missing data. Five patients in the placebo arm and 7 in the tanezumab 2.5mg arm of study 1057 were also omitted from analyses due to missing data. Nine patients were missing baseline information on race; data were imputed for these patients by assigning them to the majority race at a site. If the site had only 1 patient, they were assigned to the majority race for the entire study.

**Assessment of redundancy between selected variables**

Analyses were designed and carried out to specifically create models to examine patient variables important to WOMAC Pain responses in patients who received placebo or tanezumab. Those models were then used to identify potential subgroups of patients with an enhanced treatment effect of tanezumab versus placebo.

The matrices produced for both placebo and tanezumab 2.5mg arms showed correlations between baseline WOMAC Pain and Physical Function scores, variables associated with change from baseline in WOMAC Pain scores at week 16, insomnia and a history of psychiatric disorders, and a history of or concomitant non-OA pain (**Supplementary Figure 1**). Given the acceptably low correlation between most of the selected variables, descriptive analyses, linear models, and generalized additive models were used to describe and understand the underlying data structure.

**Supplementary Figure 1.** Correlation between patient-based variables selected for analyses.

**
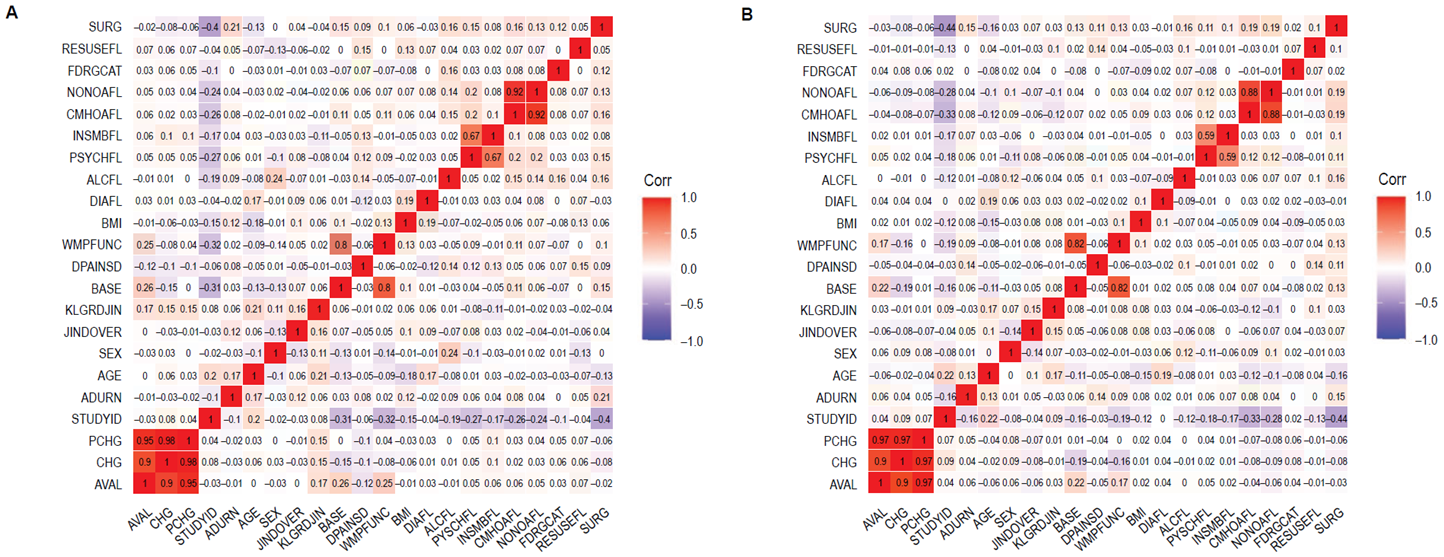
**

Spearman rank correlation matrices were used to examine the relationship between patient-based variables in the placebo (**A**) and tanezumab 2.5mg (**B**) arms. In both treatment arms, correlations were found between baseline scores for WOMAC Pain and WOMAC Physical Function, variables associated with change from baseline in WOMAC Pain scores at week 16, insomnia and a history of psychiatric disorders, and a history of or concomitant non-OA pain. Given the acceptably low correlations between most variables, analyses proceeded as planned.

Abbreviations for the variables included in the analyses are listed in Table 1 in the main article. AVAL = WOMAC Pain Score at week 16; CHG = change from baseline in WOMAC Pain score at week 16; OA = osteoarthritis; PCHG = percentage change from baseline in WOMAC Pain score at week 16; WOMAC = Western Ontario and McMaster Universities Osteoarthritis Index.

**Supplementary Figure 2.** Main effects linear regression model for variables predictive of WOMAC Pain scores at week 16 in the placebo arm.


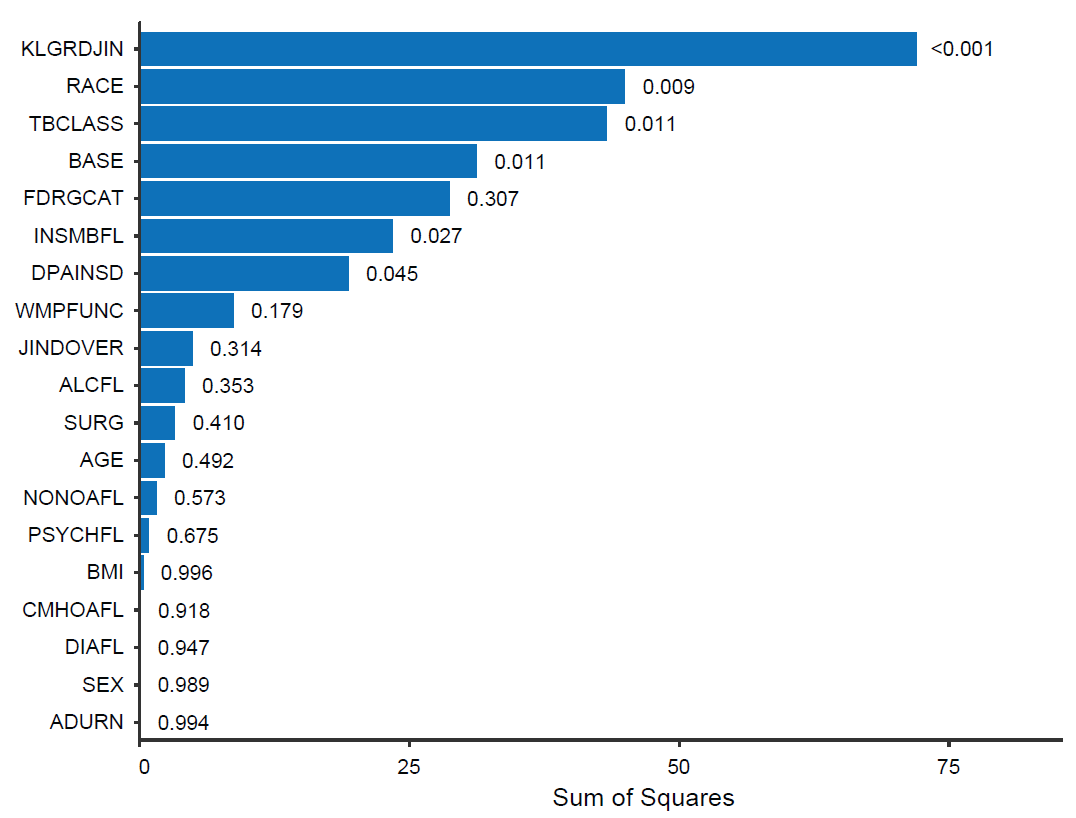


A main effects linear regression model was used to identify the variables that contributed most to WOMAC Pain scores at week 16 in patients who received placebo. In agreement with gradient boosted regression trees (**Figure 1**), the KL grade of the index joint, baseline WOMAC Pain scores, the SD of diary pain scores at baseline and baseline WOMAC Physical Function scores were identified among the most important predictors of WOMAC Pain scores at week 16. *P*-values derived from Wald tests reported only as descriptive statistics. KL = Kellgren-Lawrence; SD = standard deviation; WOMAC = Western Ontario and McMaster Universities Osteoarthritis Index. Abbreviations for the variables identified by the model are listed in Table 1.

**Supplementary Figure 3.** Marginal plots for categories of variables predictive of WOMAC Pain scores at week 16 in the placebo arm.


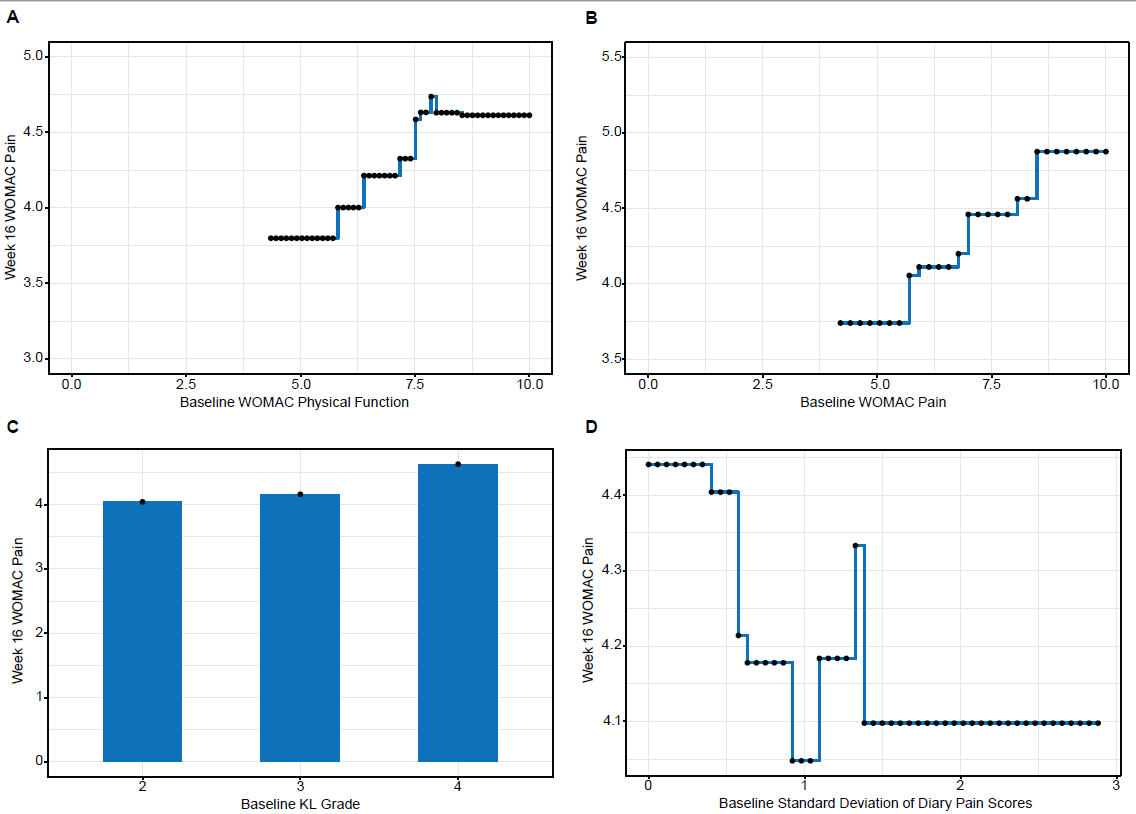


Marginal plots were used to explore the effect of each category within the variables identified as predictors of WOMAC Pain at week 16 in the placebo arm. (**A**) Patients with higher baseline WOMAC Physical Function scores had higher WOMAC Pain scores at week 16, compared with those who had lower baseline WOMAC Physical Function scores. (**B**) Patients with higher baseline WOMAC Pain scores had higher WOMAC Pain scores at week 16, compared with those who had lower baseline WOMAC Pain scores. (**C**) Patients with a KL grade of 4 in the index joint had higher WOMAC Pain scores at week 16, compared to those with grade 2 or 3. (**D**) Patients with higher standard deviations of diary pain at baseline had lower WOMAC Pain scores at week 16, compared with those with lower standard deviations of those scores.

KL = Kellgren-Lawrence; WOMAC = Western Ontario and McMaster Universities Osteoarthritis Index.

**Supplementary Figure 4.** Main effects linear regression model for variables predictive of WOMAC Pain scores at week 16 in the tanezumab 2.5mg arm.


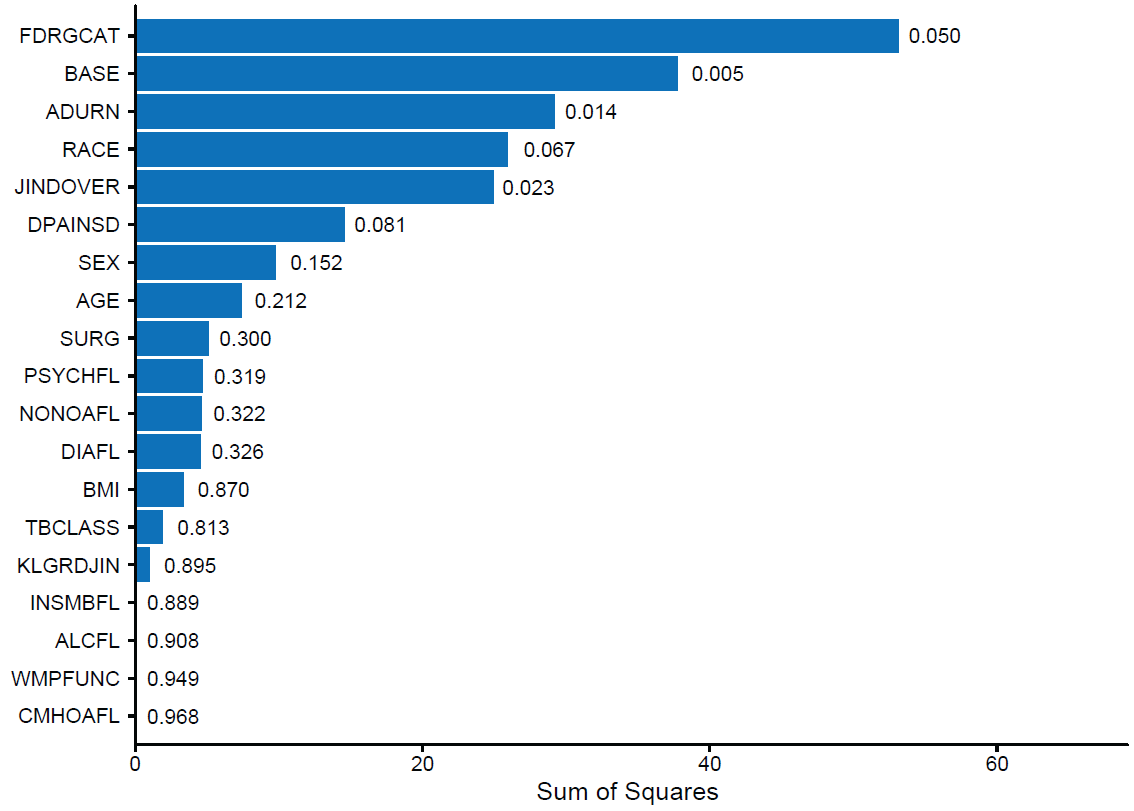


A main effects linear regression model was used to identify the variables that contributed most to WOMAC Pain scores at week 16 in patients who received tanezumab 2.5mg. Failure of prior medications, baseline WOMAC Pain scores, duration of disease and the SD of diary pain scores at baseline were identified among the most important predictors of WOMAC Pain scores at week 16.

*P*-values derived from Wald tests reported only as descriptive statistics. SD = standard deviation; WOMAC = Western Ontario and McMaster Universities Osteoarthritis Index. Abbreviations for the variables identified by the model are listed in Table 1.

**Supplementary Figure 5.** Marginal plots for categories of variables predictive of WOMAC Pain scores at week 16 in the tanezumab 2.5mg arm.


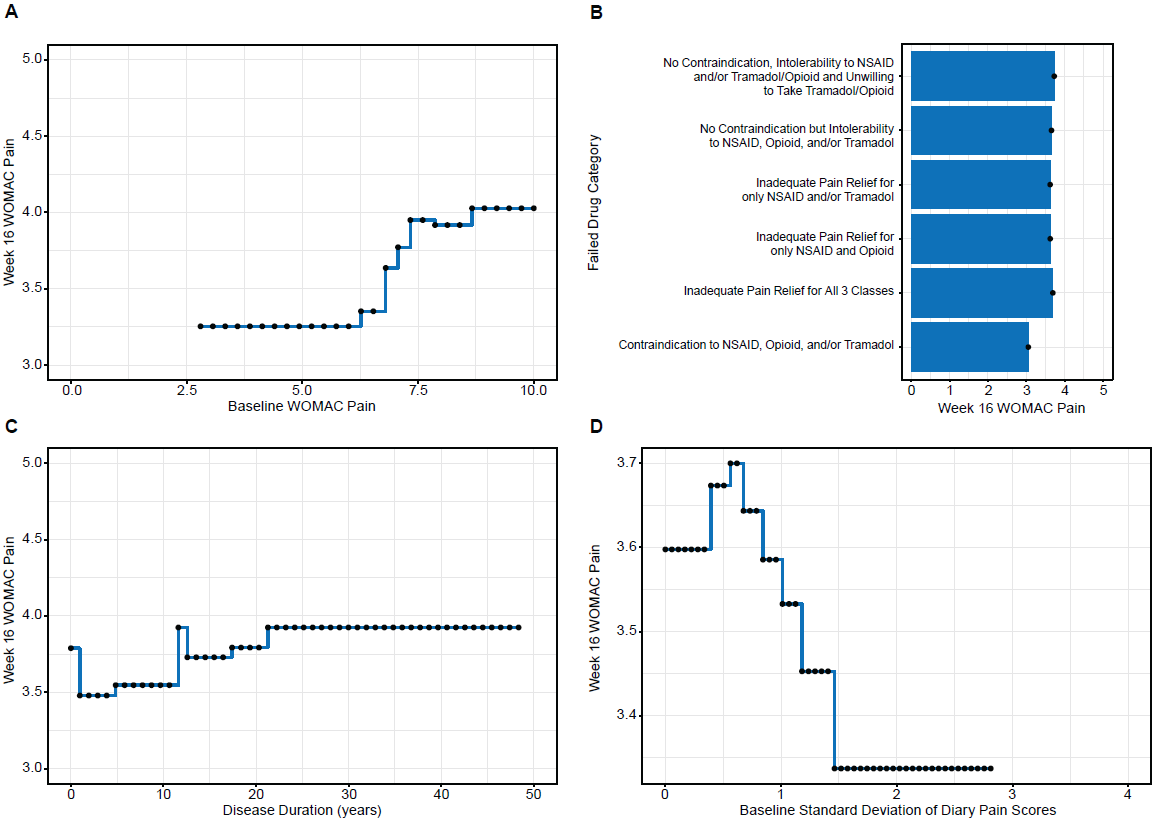


Marginal plots were used to explore the effect of each category within the variables identified as predictors of WOMAC Pain at week 16 in the tanezumab 2.5mg arm. (**A**) Patients with higher baseline WOMAC Pain scores had higher WOMAC Pain scores at week 16, compared with those who had lower baseline WOMAC Pain scores. (**B**) Patients with contraindication to NSAID, opioid, and/or tramadol had lower WOMAC Pain scores at week 16 than patients with all other classes of failure of prior medications. (**C**) With the exception of newly diagnosed patients, those with durations of disease longer than approximately 10 years had higher WOMAC Pain scores at week 16 than those with shorter durations of disease. (**D**) Patients with higher standard deviations of diary pain at baseline had lower WOMAC Pain scores at week 16, compared with those with lower standard deviations of those scores. NSAID = non-steroidal anti-inflammatory drug; WOMAC = Western Ontario and McMaster Universities Osteoarthritis Index.

**Supplementary Figure 6.** A gradient boosted regression tree fit for individual treatment effects


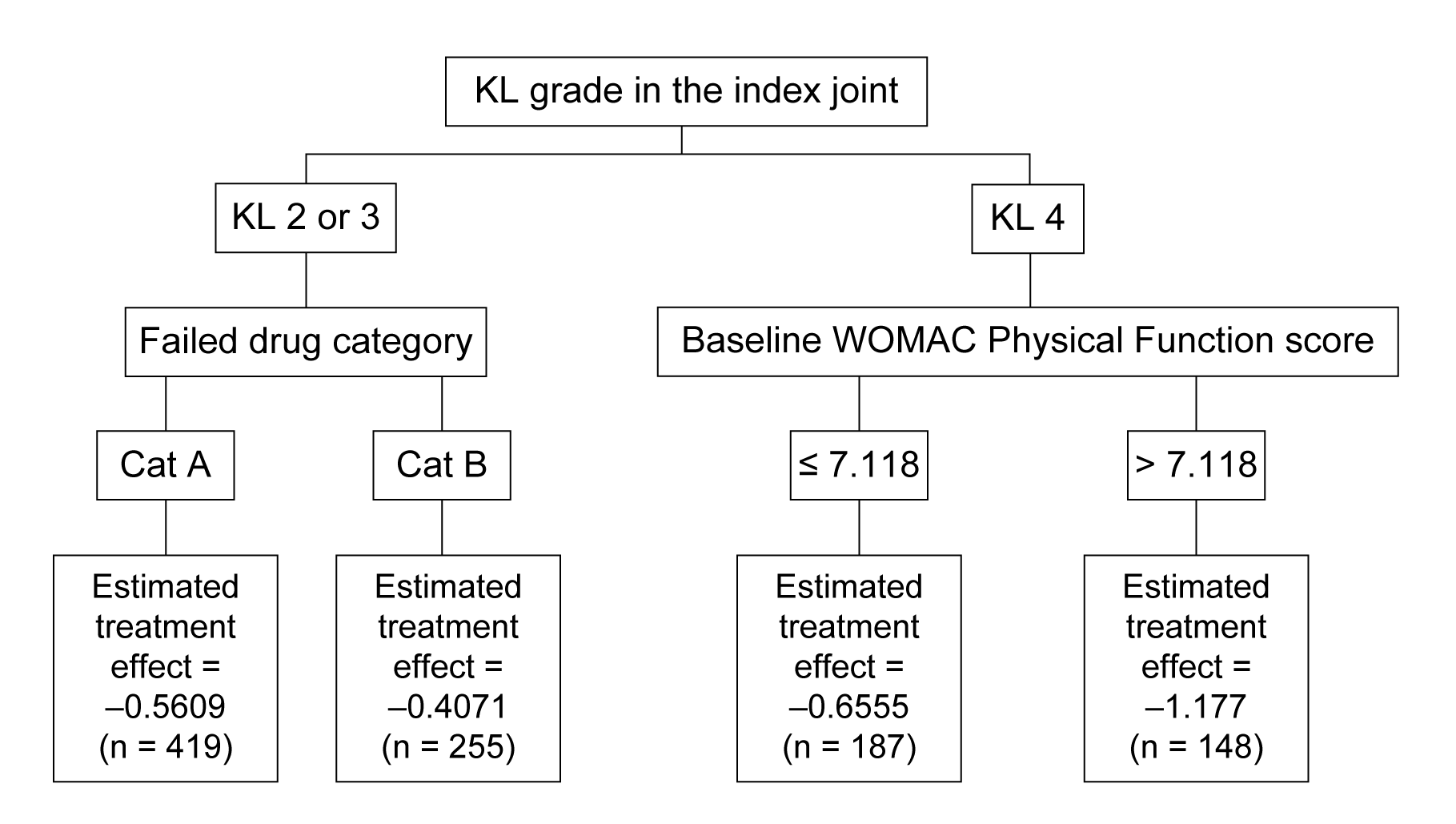


A gradient boosted regression tree fit to the calculated individual treatment effect values split the data first by KL grade of the index joint (2 or 3 versus 4). Data for patients with a KL grade of 2 or 3 were further split by failed drug category - Cat A [contraindication to NSAID, opioid, and/or tramadol, inadequate pain relief for all 3 classes, inadequate pain relief for only NSAID and/or tramadol, no contraindication but intolerability to NSAID, opioid, and/or tramadol] versus Cat B [inadequate pain relief for only NSAID and opioid, no contraindication, intolerability to NSAID and/or tramadol/opioid and unwilling to take tramadol/opioid]. Data for patients with a KL grade of 4 were further split by baseline WOMAC Physical Function score (≤7.118 versus >7.118). Within the model, patients with a KL grade of 4 and a baseline WOMAC Physical Function score of >7.118 were predicted to have an enhanced treatment effect of tanezumab versus placebo that was greater than that estimated by the linear model. Bootstrap correction of these analyses indicated that the optimism bias of the method (-0.75) was larger than enhanced treatment effect in the subgroups identified (-0.5473), suggesting that the subgroups identified did not have an enhanced treatment effect compared with the whole dataset.

**RCode for data analyses**

**# The virtual twins process first predicts a placebo and a tzb 2.5 mg**

**# pain score for each patient using the following random forest**

**# models. The input data sets for those models are plbdatgam and**

**# T25datgam. The variables used are listed in the next several lines.**

**# AVAL is Week 16 Pain Score**

**# ADURN is how long they’ve had diseage**

**# RACE, AGE, SEX, BMICAT are patient’s Race, Age, SEX, BMI category**

**# JINDOVER is the OA index joint for pain**

**# KLGRDJINFT is Kellgran-Lawrence grade for index joint**

**# BASE is baseline Womac pain**

**# DPAINSD is standard deviation of patient’s baseline pain diary**

**# scores**

**# WMPFUNC is baseline Womac function score**

**# DIAFL is diabetes indicator**

**# ALCFL is alcohol consumption indicator**

**# TBCLASS is smoking status indicator**

**# PSYCHFL is psychiatric history indicator**

**# INSMBFL is insomnia indicator**

**# CMHOAFL is concurrent non-OA medication indicator**

**# NONOAFL is history of chronic non-OA medication indicator**

**# FDRGCAT is prior treatment indicator**

**# Placebo Model**

**set.seed(1965)**

**gbplacebo16 <- randomForest(AVAL ~ RACE + ADURN + AGE + SEX + JINDOVER + KLGRDJINFT + BASE + DPAINSD + WMPFUNC + BMICAT + DIAFL +**

**ALCFL + TBCLASS + PSYCHFL + INSMBFL + CMHOAFL + NONOAFL + FDRGCAT ,**

**data=plbdatgam)**

**gbplacebo16**

**# Tanezumab Model**

**set.seed(1965)**

**gbT2516 <- randomForest(AVAL ~ RACE + ADURN + AGE + SEX +**

**JINDOVER + KLGRDJINFT + BASE + DPAINSD + WMPFUNC + BMICAT + DIAFL + ALCFL + TBCLASS + PSYCHFL + INSMBFL + CMHOAFL + NONOAFL + FDRGCAT ,**

**data=T25datgam)**

**gbT2516**

**# The second step in the process calculates differences in virtual**

**# twins, by computing the fitted values under treatment and placebo**

**# for ALL subjects**

**vtbothplb <- bothdatgam %>% mutate(TRTP= factor(x="Placebo" , levels= c("Placebo","Tanezumab 2.5 mg")) )**

**vtbothtnz <- bothdatgam %>% mutate(TRTP= factor(x="Tanezumab 2.5 mg" , levels= c("Placebo","Tanezumab 2.5 mg")) )**

**pred.plb <- predict(gbplacebo16, newdata = vtbothplb)**

**pred.tnz <- predict(gbT2516, newdata = vtbothtnz)**

**# The following combines all data and predictions for input to**

**# a tree building algorithm and plotting of the results.**

**augall <- bind_cols( data.frame(pred.plb, pred.tnz), vtboth) %>%**

**mutate(ite=pred.tnz-pred.plb)**

**moborig16 <- glmtree(formula = ite ~ RACE + ADURN + AGE + SEX +**

**JINDOVER + KLGRDJINFT +BASE + DPAINSD + WMPFUNC + BMICAT + DIAFL +**

**ALCFL + TBCLASS + PSYCHFL + INSMBFL + CMHOAFL + NONOAFL + FDRGCAT ,**

**data=augall, verbose = F, bonferroni=T,**

**minsize = 50, maxdepth =3, alpha = .05, prune = "BIC",**

**family =gaussian)**

**print(moborig16)**

**plot(moborig16, terminal_panel=NULL)**

**plottree <- ggparty(moborig16) +**

**geom_edge() +**

**geom_edge_label(id=c(1,2,4,5,6), size=3) +**

**geom_node_label(aes(label=splitvar), ids="inner")+**

**geom_node_plot( gglist=list(geom_boxplot(aes(y=ite))) ) +**

**ggtitle("Virtual Twins: Week 16 WOMAC Score")**

**plottree**
